# Differential Modulation of GABA_A_ Receptors Underlies Postsynaptic Depolarization- and Purinoceptor-Mediated Enhancement of Cerebellar Inhibitory Transmission: A Non-Stationary Fluctuation Analysis Study

**DOI:** 10.1371/journal.pone.0150636

**Published:** 2016-03-01

**Authors:** Yumie Ono, Fumihito Saitow, Shiro Konishi

**Affiliations:** 1 Department of Electronics and Bioinformatics, School of Science and Technology, Meiji University, Room A806, 1-1-1 Higashi-Mita, Tama-ku, Kawasaki, Kanagawa, 214–8571, Japan; 2 Department of Pharmacology, Nippon Medical School, 1-1-5 Sendagi, Bunkyo-ku, Tokyo 113–8602, Japan; 3 Department of Neurophysiology, Kagawa School of Pharmaceutical Sciences and Institute of Neuroscience, Tokushima Bunri University, 1314–1 Shido, Sanuki-shi, Kagawa 769–2193, Japan; Tokai University, JAPAN

## Abstract

Cerebellar GABAergic inhibitory transmission between interneurons and Purkinje cells (PCs) undergoes a long-lasting enhancement following different stimulations, such as brief depolarization or activation of purinergic receptors of postsynaptic PCs. The underlying mechanisms, however, are not completely understood. Using a peak-scaled non-stationary fluctuation analysis, we therefore aimed at characterizing changes in the electrophysiological properties of GABA_A_ receptors in PCs of rat cerebellar cortex during depolarization-induced “rebound potentiation (RP)” and purinoceptor-mediated long-term potentiation (PM-LTP), because both RP and PM-LTP likely depend on postsynaptic mechanisms. Stimulation-evoked inhibitory postsynaptic currents (eIPSCs) were recorded from PCs in neonatal rat cerebellar slices. Our analysis showed that postsynaptic membrane depolarization induced RP of eIPSCs in association with significant increase in the number of synaptic GABA_A_ receptors without changing the channel conductance. By contrast, bath application of ATP induced PM-LTP of eIPSCs with a significant increase of the channel conductance of GABA_A_ receptors without affecting the receptor number. Pretreatment with protein kinase A (PKA) inhibitors, H-89 and cAMPS-Rp, completely abolished the PM-LTP. The CaMKII inhibitor KN-62 reported to abolish RP did not alter PM-LTP. These results suggest that the signaling mechanism underlying PM-LTP could involve ATP-induced phosphorylation of synaptic GABA_A_ receptors, thereby resulting in upregulation of the channel conductance by stimulating adenylyl cyclase-PKA signaling cascade, possibly via activation of P2Y_11_ purinoceptor. Thus, our findings reveal that postsynaptic GABA_A_ receptors at the interneuron-PC inhibitory synapses are under the control of two distinct forms of long-term potentiation linked with different second messenger cascades.

## Introduction

Synaptic plasticity, such as long-term potentiation (LTP) or long-term depression, is a potential cellular basis of learning and memory. Extensive studies have revealed the underlying mechanisms of the synaptic plasticity at AMPA receptor-mediated excitatory synapses, however, relatively little is known about those at inhibitory synapses. A synaptic process termed rebound potentiation (RP), found in GABAergic inhibitory synapses between cerebellar interneurons and Purkinje cells (PCs), is one form of long-term upregulation of GABA_A_ receptor-mediated transmission induced by direct depolarization of the postsynaptic PC [1]. Although the increase of intracellular Ca^2+^ concentration and subsequent activation of Ca^2+^/calmodulin-dependent protein kinase II (CaMKII) appear to mediate the induction and maintenance of RP [2, 3], the mechanism by which CaMKII induces RP remains elusive. In the LTP at excitatory glutamatergic synapses, CaMKII is reported to affect AMPA receptors via at least two distinct mechanisms. CaMKII directly phosphorylates GluR1 subunit at Ser831 to increase the conductance of existing AMPA receptors [4, 5]. Alternatively, CaMKII appears to indirectly cause insertion of new AMPA receptors into the postsynaptic membrane, likely through phosphorylation of a PDZ domain or GTPase-activating protein [6, 7]. Lüthi *et al*. [8] reported that among hippocampal CA1 pyramidal cells that developed LTP and the subsequent depotentiation, only 60% of them were associated with an alternation in receptor conductance. These findings suggest that distinct mechanisms of synaptic plasticity are separately involved in LTP at glutamatergic excitatory synapses and prompted us to examine what molecular mechanisms underlie the upregulation of GABA_A_ receptors during RP.

In addition to RP, it has recently been demonstrated that another form of synaptic plasticity operates in response to activation of purinoceptors at cerebellar GABAergic synapses where both ionotropic P2X and metabotropic P2Y receptors are implicated in presynaptic and postsynaptic enhancement of GABAergic transmission onto PCs [9–12]. Saitow *et al*. [10] found that ATP and its analogs induced an enhancement of stimulation-evoked inhibitory postsynaptic currents (eIPSCs) recorded in PCs through activation of postsynaptic P2Y receptors, outlasting tens of minutes after washout of P2Y receptor agonists, which was hereinafter referred to as purinoceptor-mediated long-term potentiation (PM-LTP). Even though PM-LTP requires elevation of intracellular Ca^2+^ as RP does, the time course of PM-LTP is much slower than that of RP and is independent of influx of extracellular Ca^2+^ that is essential for induction of RP. These observations raise the possibility that distinct mechanisms underlie the enhancement of the GABA_A_ receptor sensitivity during RP and PM-LTP induced in PCs.

Peak-scaled non-stationary fluctuation analysis (PS-NSFA) could characterize the properties of single receptor channels, the number of active receptors and the single channel current, based on fluctuations detected during synaptic responses. Since PS-NSFA and its analogous fluctuation analysis have been widely used to investigate the electrophysiological properties at glutamatergic as well as GABAergic synapses [13, 14], we adopted this analysis to examine how GABA_A_ receptors in PCs could be modulated during RP and PM-LTP. We also performed pharmacological experiments where the effects of protein kinase inhibitors were tested to further explore signaling mechanisms underlying PM-LTP.

## Materials and Methods

All experiments were carried out in accordance with the National Institutes of Health (NIH) and National Advisory Committee for Laboratory Animal Research (NACLAR) guidelines, and approved by the Biopolis Institutional Animal Care and Use Committee.

### Slice preparation

Wistar rats of both sexes (9- to 14-day old) were deeply anesthetized with halothane, and the brain was quickly removed from the skull and immersed in an ice-cold Na^+^-deficient saline containing (in mM): 299.2 sucrose, 3.4 KCl, 0.3 CaCl_2_, 3.0 MgCl_2_, 10.0 HEPES, 0.6 NaH_2_PO_4_, and 10.0 glucose. A block of the cerebellum was placed on a microslicer (VT 1000S, Leica, Nussloch, Germany) and cut into parasagittal slices (250 μm thick). Slices were kept more than 1 h in a humidified and oxygenated chamber filled with a modified artificial cerebrospinal fluid (ACSF) containing (in mM): 138.6 NaCl, 3.35 KCl, 2.5 CaCl_2_, 1.0 MgCl_2_, 21.0 NaHCO_3_, 0.6 NaH_2_PO_4_, and 10.0 glucose (pH 7.4, equilibrated with 95% O_2_−5% CO_2_ gas).

### Patch-clamp recording and stimulation

A cerebellar slice was placed on the bottom of the recording chamber attached on the stage of a microscope (BX-51WI, Olympus, Tokyo, Japan) and held in position by a nylon mesh. Patch clamp electrodes were pulled from borosilicate glass tubing (GD-1.5, Narishige, Tokyo, Japan) and filled with an internal solution containing (in mM): 78.0 CsCl, 64.0 Cs methanesulfonate, 1.0 MgCl_2_, 1.0 K-EGTA, 10.0 Na-HEPES, 2.0 QX-314, 3.0 Mg-ATP, and 0.4 Na-GTP, pH 7.4. Resistance of the electrodes was 2−4 MΩ. Whole-cell patch clamp recordings were obtained from PCs that were visually identified under the infrared differential interference contrast (IR-DIC) optics with a water immersion objective lens (60×, NA 0.90, Olympus) and an IR-CCD camera (C2741-79H, Hamamatsu Photonics, Hamamatsu, Japan). Membrane currents and potentials were recorded with a patch-clamp amplifier (EPC-8; HEKA Electronik, Lambrecht, Germany). All signals filtered at 3 kHz and sampled at 5 kHz were stored in a computer for off-line analysis. Synaptic responses were recorded at 24−26°C in the chamber continuously perfused with the oxygenated ACSF at a flow rate of 1.0 ml/min, and 10 μM 2,3-Dioxo-6-nitro-1,2,3,4- tetrahydrobenzo[f]quinoxaline-7-sulfonamide (NBQX) was added into ACSF to block ionotropic glutamate receptor-mediated excitatory synaptic currents.

To evoke IPSCs, basket cell axons were stimulated every 15 sec by a paired pulse (50 μs and 0.12 mA) via a glass microelectrode filled with ASCF and placed in the molecular layer. RP was induced by injecting ten depolarizing pulses (from –70 to 0 mV at 1 Hz for 500 msec) into PCs. PM-LTP was induced by bath application of 100 μM adenosine 5’-triphosphate (ATP) for 5 min. We chose ATP as a purinoceptor agonist because it could act as an endogenous purinergic agonist and has been shown to be co-released with GABA from hypothalamic and spinal cord neurons [15, 16]. In the cerebellar cortex, ATP could be released from interneurons in the molecular layer [17] and evoke a Ca^2+^ increase in Bergmann glial cells [18]. There has also been accumulating evidence that ATP could serve as a gliotransmitter released from astrocytes at tetrapartite synapses [19, 20].

### Peak-scaled non-stationary fluctuation analysis (PS-NSFA)

Using PS-NSFA [13, 21–26], eIPSCs were analyzed to estimate changes in the number (*N*) or single-channel current (*i*) of functional GABA_A_ receptors before and after induction of RP or PM-LTP. Briefly, IPSC waveforms were aligned with the time point of maximal rise, estimated by 5-point moving average, and averaged. The averaged mean-current waveform was scaled to the peak amplitude of individual IPSCs, subtracted and squared. The variance of the fluctuation around the mean-current was calculated for 30 bins of equal current decrement from the peak to 5 times of half decay time of the mean-current. The binned variance, averaged through all the eIPSCs, was plotted versus the mean-current amplitude. The *N* and *i* values were estimated by least-square fitting of the peak scaled variance and mean-current curve to the theoretical relationship:

$$\sigma^{2}=iI-I^{2}/N+b$$

where *σ*^2^ is the variance, *I* is the mean-current, and *b* is baseline variance. In this study, more than 20 eIPSCs without spontaneous IPSC overlap were carefully selected for each analysis. We used *N* and *i* values calculated from eIPSCs recorded during 10 minutes just before injection of depolarization pulses or application of ATP as baseline (control). The *N* and *i* values calculated from eIPSCs recorded between 10 and 20 min after membrane depolarization or ATP application, during which the amplitude and the decay time of IPSCs reached the plateau, provided the basis for our statistical comparisons. All the analysis was done using MATLAB software (MathWorks, MA, USA).

### Validation of PS-NSFA

**Fig 1:**
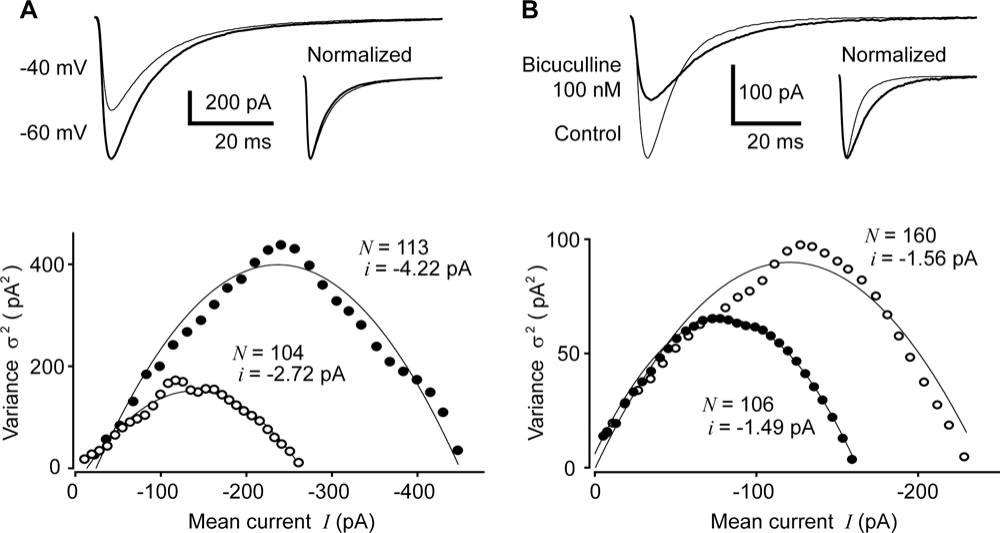
Validation of PS-NSFA applied to eIPSCs at GABAergic synapses between basket cells and PCs. (A) Effects of changing the driving force for GABA_A_ receptor activation on eIPSCs. Upper panel: averaged traces of eIPSCs recorded at the holding membrane potential of –40 mV (thin line) and -60 mV (thick line). Inset shows the averaged trace of IPSCs recorded at −40 mV was scaled to the same amplitude of those recorded at –60 mV (Normalized). Lower panel: mean-variance curves at the membrane potentials of –40 (open circles) and –60 mV (closed circles). *N* and *i* indicate the number of active GABA_A_ receptors and the size of unitary current through GABA_A_ receptors estimated from the mean-variance curves, respectively. (B) Effects of changing the GABA_A_ receptor availability by bicuculline on eIPSCs. Upper panel: averaged traces of eIPSCs before (control) and after bath-application of the GABA_A_ receptor antagonist bicuculline (100 nM). Inset shows averaged trace of IPSCs in the presence of bicuculline was scaled to the same amplitude of those recorded in control solution (Normalized). Note that the decay time was prolonged after partial blockade of GABA_A_ receptors by bicuculline. Lower panel: mean-variance curves before (open circles) and after (closed circles) application of bicuculline. *N* and *i* indicate the number of active GABA_A_ receptors and the size of unitary current through the GABA_A_ receptor estimated from the mean-variance curves, respectively.

To check the validity of our PS-NSFA in evaluating GABA_A_ receptor characteristics of eIPSCs recorded at interneuron-PC inhibitory synapses, we first conducted two types of control experiments, in which we attempted to change the unitary current and the number of GABA_A_ receptors, respectively, by changing the membrane potential and by applying the GABA_A_ receptor antagonist bicuculline (Fig 1). Shifting the holding potential, namely changing the driving force for activation of GABA_A_ receptors affected only the size of unitary current through GABA_A_ receptors, whereas the number of channels remained almost constant. The slope near *I* = 0, approximately corresponding to *i*, was increased with the increment of membrane potential (Fig 1A). In contrast, estimated number of receptor channels was reduced when GABA_A_ receptors were partially blocked by a low concentration of bicuculline (100 nM), but the unitary current amplitudes remained comparable before and after the bicuculline treatment. Accordingly, the curve height decreased as bicuculline reduced the number of active receptor channels, whereas the slope is unaffected, indicating that the intensity of unitary current passing through GABA_A_ receptors was not affected by bicuculline (Fig 1B).

### Drug application

ATP disodium salt, cAMPS-Rp triethylammonium salt (Rp-Adenosine, 3′, 5′-cyclic monophosphorothioate), H-89 and KN-62 were obtained from Sigma (Singapore). NBQX was obtained from Tocris Cookson (Bristol, UK). All the drugs were dissolved in ACSF and applied by perfusion. In experiments for testing the effects of H-89, cAMPS-Rp or KN-62, we recorded control eIPSCs in the presence of each one of inhibitors for more than 10 minutes before application of ATP to confirm that there were no significant changes in the amplitude, rise time and decay time of eIPSC.

### Statistics

Numerical data are presented as mean ± S.E.M., and statistical significance was assessed by Student’s paired or unpaired t-test. The p-value was indicated as *p*. We evaluated changes in the mean amplitude of eIPSCs by comparing the values obtained during the periods of 10 to 15 minutes after membrane depolarization or ATP application and those obtained during 5 minutes of control conditions. In experiments for testing the effects of H-89, cAMPS-Rp or KN-62, Dunnett’s multiple comparison test was further conducted to compare the differences in the eIPSC amplitude or parameters of PS-NSFA among individual experimental groups for pre- and post-ATP application periods.

## Results

### Long-term enhancement of inhibitory interneuron—Purkinje cell synapses by membrane depolarization and ATP application

**Fig 2:**
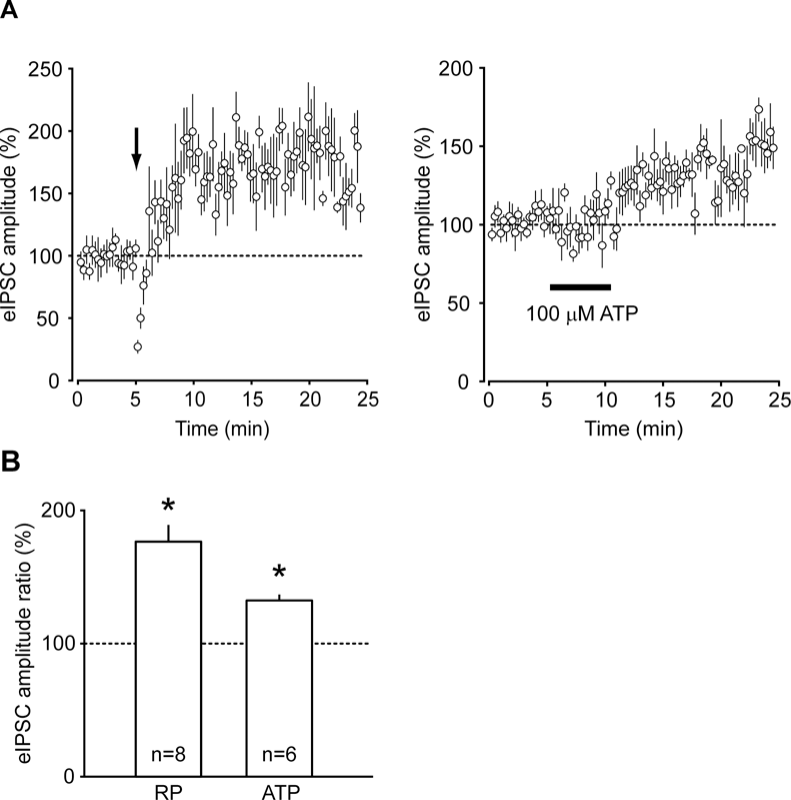
Rebound potentiation (RP) and purinoceptor-mediated long-term potentiation (PM-LTP) of eIPSCs at basket cell-PC synapses. (A) Time course changes of eIPSC amplitude in the course of RP (n = 8, left panel) and PM-LTP (n = 6, right panel). RP was induced by depolarization pulses given to individual PCs at the time point indicated by an arrow. PM-LTP was induced by bath-application of 100 μM ATP during the period of 5 min indicated by a horizontal bar. (B) Mean amplitude of eIPSCs during the period of 10 to 15 min after induction of RP or PM-LTP. Asterisk indicates a statistically significant difference compared to mean amplitude of eIPSCs before induction of RP or PM-LTP (p<0.01).

Membrane depolarization induced RP of eIPSCs with a significant increase in the amplitude to 176.5 ± 12.5% of control (Fig 2A, n = 8, *p*<0.001). In agreement with earlier findings [3], the augmentation of eIPSCs reached the plateau within 5 min after membrane depolarization, persisting for at least 20 min. Application of 100 μM ATP also significantly increased the amplitude of eIPSCs to 132.3 ± 4.4% of control (Fig 2B, n = 6, *p* < 0.001). In contrast to RP, the amplitude of eIPSCs showed gradual increase that lasted for more than 10 min after administration of ATP. The PM-LTP was preceded by transient increase in amplitude and frequency of spontaneous IPSC during ATP application (data not shown). The gradual increase of eIPSCs and the short-term enhancement of spontaneous IPSCs preceding PM-LTP were consistent with the previous reports [9, 10, 27].

### Increased number of GABA_A_ receptors in RP and increased single-channel current in PM-LTP

**Fig 3:**
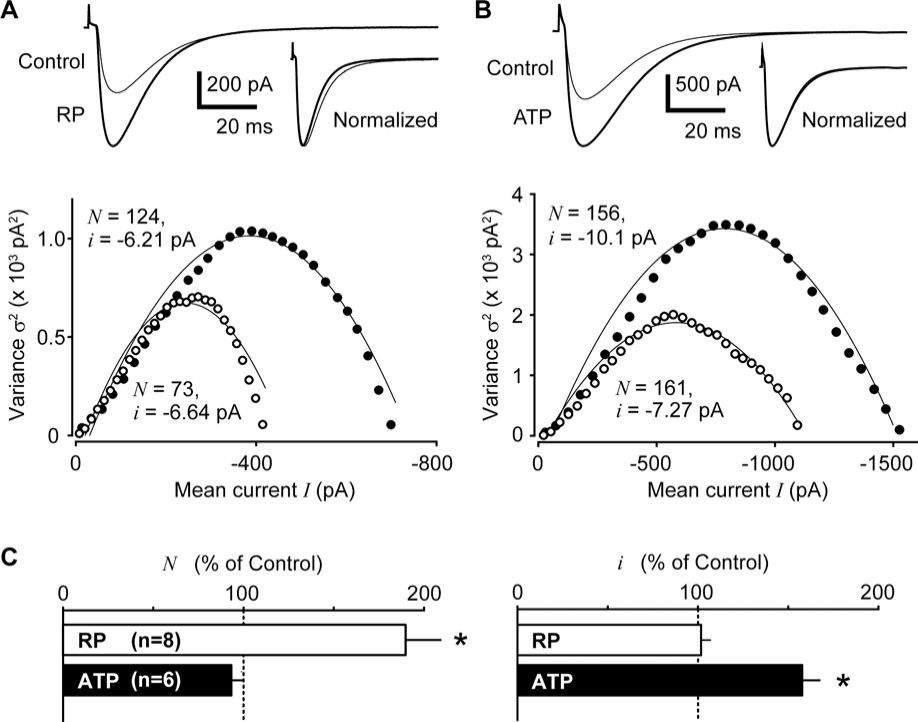
Two distinct underlying mechanisms revealed by the PS-NSFA method: RP and PM-LTP were associated with increases in the number of active GABA_A_ receptors and in the intensity of single channel conductance, respectively. (A) PS-NSFA before and during RP. Upper panel: representative averaged traces of eIPSCs recorded before (thin line: Control) and after induction of RP (thick line: RP). Inset shows averaged trace of IPSCs recorded during the control period was scaled to the same amplitude of those recorded during RP (Normalized). Lower panel: representative plots of mean-variance curves before (control, open circles) and after induction of RP (closed circles). (B) PS-NSFA before and during PM-LTP. Upper panel: representative averaged traces of eIPSCs before (thin line: Control) and after application of ATP (thick line: ATP). Inset shows averaged trace of IPSCs recorded during the control period was scaled to the same amplitude of those recorded during PM-LTP induced by ATP (Normalized). Lower panel: representative plots of mean-variance curves before (control, open circles) and after induction of PM-LTP (closed circles). (C) Comparison of the mean value changes in the number of functioning GABA_A_ receptors (*N*) and the size of unitary current through the GABA_A_ receptor (*i*) during RP and ATP-induced PM-LTP. Asterisk indicates a statistically significant difference (p<0.01).

Fig 3A and 3B show typical eIPSCs recorded during RP and PM-LTP. Rise time (10 to 90% of the peak amplitude) of eIPSCs was not altered in both RP and PM-LTP. The mean rise time was 3.7 ± 0.3 ms and 3.3 ± 0.2 ms (n = 8, *p* = 0.07) before and after membrane depolarization, and 3.3 ± 0.2 ms and 3.1 ± 0.2 ms (n = 6, *p* = 0.18) before and after ATP application, respectively. There was, however, significant reduction in the decay time (90 to 10% of the peak amplitude) of eIPSCs after membrane depolarization (Fig 3A, upper panel), but not after ATP application (see Discussion). The mean decay time were 32.2 ± 2.3 ms and 28.2 ± 1.7 ms (n = 8, *p* < 0.05) before and after membrane depolarization, respectively, whereas those remained unaltered (21.6 ± 9.5 ms and 21.4 ± 9.6 ms, *p* = 0.72) before and after application of ATP, respectively. These results indicate that the electrophysiological properties of GABAergic transmission are differentially modulated during RP and PM-LTP.

As illustrated in Fig 3, we further analyzed eIPSCs by PS-NSFA to characterize the changes in the single channel properties following membrane depolarization or ATP application. During RP, the number of functioning GABA_A_ receptors (*N*) significantly increased to 189.7 ± 19.8% of control condition (n = 8, *p* < 0.01), while the single-channel current (*i*) was not altered (102.2 ± 5.5%, n = 8, *p* = 0.697, Fig 3A and 3C). Interestingly, the number of GABA_A_ receptors remained unchanged during PM-LTP (93.3 ± 6.3%, n = 6, *p* = 0.333), while the single-channel current significantly increased to 158.7 ± 10.1% (n = 6, *p* < 0.01, Fig 3B and 3C). These results indicate that RP could be induced mainly by insertion of novel functioning GABA_A_ receptors into interneuron-PC inhibitory synapses, whereas the upregulation of membrane conductance of existing GABA_A_ receptors dominantly contributes to the induction of PM-LTP.

### PM-LTP involves PKA-dependent signaling pathway

**Fig 4:**
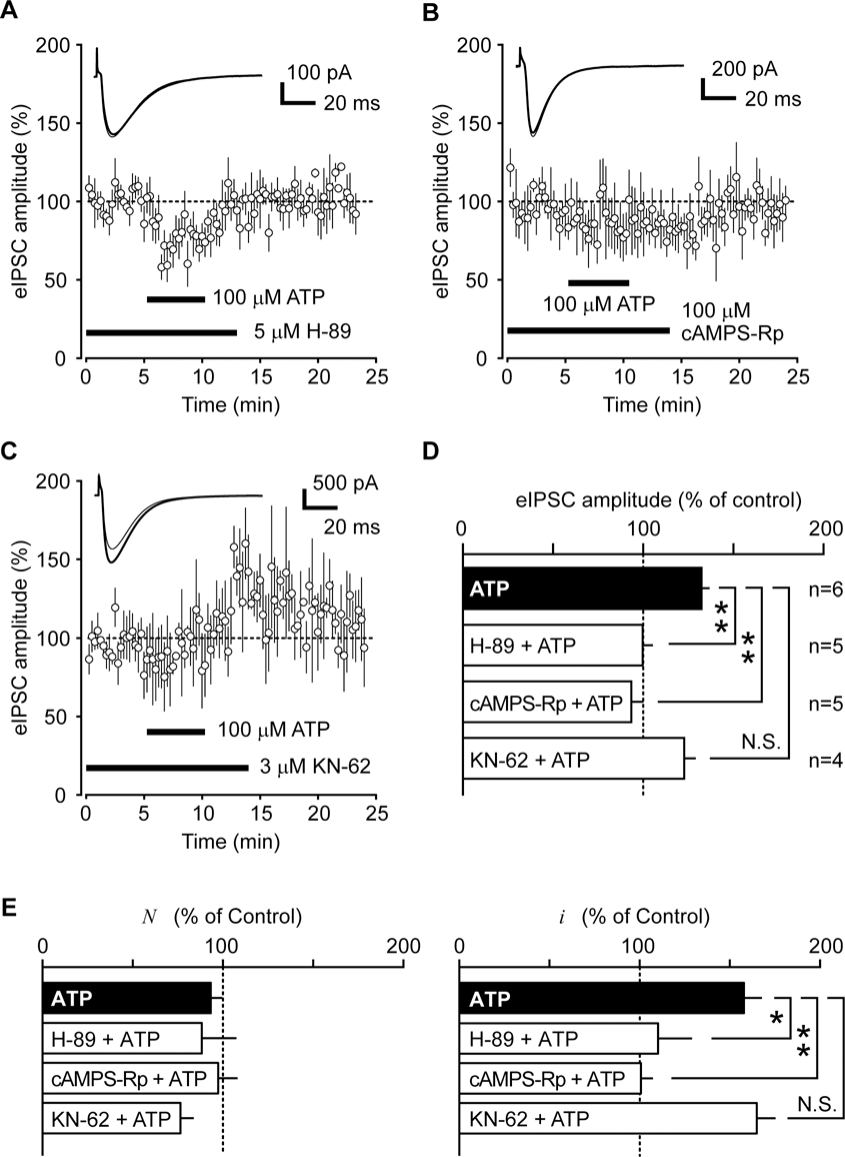
Effects of PKA and CaMKII inhibitors on PM-LTP. (A, B,C) Time course change of the eIPSC amplitudes in experiments where ATP (100 μM) was applied after treatments with PKA inhibitors, H-89 (A) and cAMPS-Rp (B), and a CaMKII inhibitor, KN-62 (C). Inset shows representative averaged traces of eIPSC recorded before (thin line) and after ATP application (thick line). (D) Effects of PKA and CaMKII inhibitors on the mean amplitudes of eIPSCs recorded during the periods10 to 15 min after ATP application. (E) Effects PKA and CaMKII inhibitors on the mean values of changes in the number of functioning GABA_A_ receptors (*N*) and the size of unitary current through the GABA_A_ receptor (*i*). Filled bars (ATP) indicate the values obtained after application of ATP alone (data taken from Figs 2B and 3C). Asterisk indicates a level of statistically significant difference (*: p<0.05, **: p<0.01, N.S.: no significant difference).

Since our data from PS-NFSA indicated that RP and PM-LTP depend on different modes of modulation leading to the increases in the activity of GABA_A_ receptors, we then sought to examine whether or not PM-LTP could recruit the second messenger signaling cascade involving CaMKII for its mechanism of induction and maintenance, as has been reported for RP [28, 29]. To ask this question, we examined the effects of a CaMKII inhibitor, KN-62, and PKA inhibitors, H-89 and cAMPS-Rp, on PM-LTP. Interestingly, the CaMKII inhibitor KN-62 failed to prevent the induction of PM-LTP. The amplitude of eIPSCs recorded during 10 to 15 min after ATP (100 μM) application significantly increased to 122.7 ± 6.5% of control (n = 4, *p* = 0.55 vs. application of ATP alone, 132.3 ± 4.4%, n = 6, Dunnett test, Fig 4C and 4D) in the presence of KN-62 (3 μM). Furthermore, after treatment with KN-62, ATP did not significantly alter the number of GABA_A_ receptors (76.8 ± 7.4%, n = 4, *p* = 0.68 vs. application of ATP alone, 93.3 ± 6.3%, n = 6, Dunnett test), while the single-channel current was increased to 165.7 ± 10.4% (n = 4, p = 0.96, Dunnett test vs. application of ATP alone, 158.6 ± 10.1%, n = 6, Fig 4E). However, the amplitude of eIPSCs declined to the control level during the period 15 to 20 min after application of ATP (110.0 ± 10.6%, n = 4).

In contrast, treatment with the PKA inhibitors completely suppressed PM-LTP. ATP application did not affect the amplitude of eIPSCs in the presence of either H-89 (5 μM; 99.6 ± 5.7% of control, n = 5, *p* = 0.002 vs. application of ATP alone, Dunnett test, Fig 4A and 4D) or cAMPS-Rp (100 μM; 93.4 ± 6.8% of control, n = 5, *p* = 4×10^−4^ vs. application of ATP alone, Dunnett test, Fig 4B and 4D). Neither the number of GABA_A_ channels (88.3 ± 19.2% and 97.2 ± 10.9% of control in H-89 and cAMPS-Rp, n = 5 each, respectively) nor the single-channel current (110.9 ± 18.9% and 101.2 ± 6.8% of control in H-89 and cAMPS-Rp, n = 5 each, respectively) was significantly altered by ATP application after the treatment with either H-89 or cAMPS-Rp (Fig 4E).

## Discussion

In this study, we found that: (1) depolarization-induced RP of GABAergic transmission at cerebellar interneuron-PC inhibitory synapses could be caused by an increase in the number of synaptic GABA_A_ receptors without changes in the receptor channel conductance; and (2) activation of purinoceptors by ATP resulted in another form of plasticity, PM-LTP, at the GABAergic synapses through an increment of GABA_A_ receptor conductance without discernible changes in the receptor channel number. Moreover, the PKA inhibitors H-89 and cAMPS-Rp, but not the CaMKII inhibitor KN-62, completely suppressed the induction of PM-LTP, suggesting that PM-LTP involves the mobilization of PKA-dependent signaling pathways for its induction. Our results are in a sharp contrast to the previous findings that CaMKII-dependent signaling pathways underlie the induction and maintenance of RP. Taken together, it is therefore highly likely that cerebellar interneuron-PC inhibitory synapses undergo profound synaptic plasticity caused by at least two distinct postsynaptic mechanisms: one is PM-LTP, namely ATP-induced enhancement of GABAergic transmission, that could be mediated by cAMP-PKA-dependent upregulation of single GABA_A_ receptor channel conductance in PCs, and the other RP, namely postsynaptic depolarization-induced enhancement of GABAergic transmission, that could recruit the insertion of novel GABA_A_ receptors into the synaptic sites between interneurons and PCs.

**Fig 5:**
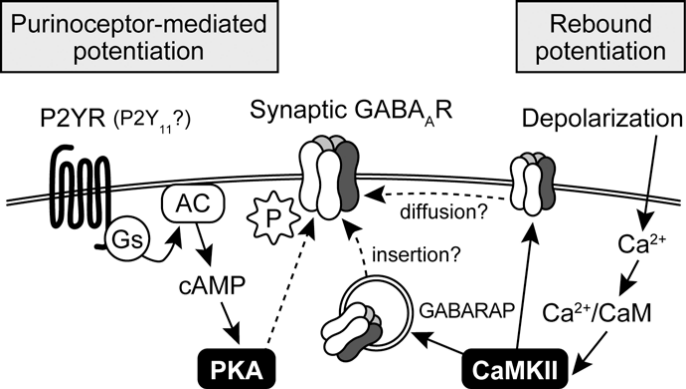
Schematic representation of different signaling cascades involved in the mechanisms underlying RP and PM-LTP. AC, adenylyl cyclase; CaM, Calmodulin; GABARAP, GABA_A_ receptor-associated protein; P, phosphate.

RP has been reported to depend on Ca^2+^ influx via voltage-gated Ca^2+^ channels [2], leading to activation of CaMKII [28, 29]. Our data from PS-NSFA provide a hint for downstream of CaMKII activation: (1) although β1 and γ2 subunits of GABA_A_ receptors have specific phosphorylation sites for CaMKII [30, 31], CaMKII activation at cerebellar GABAergic synapses unlikely couples to direct phosphorylation of synaptic GABA_A_ receptors, and (2) a possible target of CaMKII activation could be molecular component(s) involved in GABA_A_ receptor trafficking into the inhibitory synaptic sites. Consistent with this notion is the finding by Kawaguchi and Hirano [32] that GABA_A_ receptor-associated protein (GABARAP) plays a critical role in RP by its binding to GABA_A_ receptor γ2 subunit and tubulin in a manner depending on CaMKII activation. GABARAP has been implicated in the mobilization of clustering and the cell surface expression of γ2-containing GABA_A_ receptors [33]. The fact that membrane depolarization does not alter the amount of surface GABA_A_ receptors in PCs [32] suggests that GABARAP likely increases the number of synaptic GABA_A_ receptors by increasing their local diffusion from extrasynaptic to synaptic site [34, 35]. Taken together, the mechanism underlying RP could be triggered by membrane depolarization-derived Ca^2+^ influx via voltage-gated Ca^2+^ channels that could activate CaMKII, coupling to stimulation of GABARAP, thereby increasing the number of synaptic GABA_A_ receptors, possibly via lateral diffusion of extrasynaptic GABA_A_ receptors (Fig 5).

The observation in this study that the decay time of eIPSCs was reduced during the postsynaptic depolarization-induced RP (see the inset traces in Fig 3A) could be explained by the possibility that depolarization might cause liberation of retrograde messengers that could act on BC (basket cell) nerve terminals, thereby changing the kinetics of GABA release and affecting the decay time of eIPSCs. Moreover, the time course of eIPSCs was prolonged in the presence of bicuculline (see Fig 1B, upper traces). It might be conceivable that bicuculline might remove some GABAergic inhibitory effects on BCs and/or GABA release, thereby prolonging the sIPSC time-course and that the binding of bicuculline to GABA_A_ receptors may result in alteration of kinetics of receptor channel opening and closing. Although these changes in eIPSC kinetics are intriguing, it seems however unfeasible to further determine what mechanisms underlie these effects caused by RP and bicuculline.

The present finding that activation of purinoceptors by ATP was associated with long-term enhancement of the GABA_A_ receptor unitary current, but unlikely linked with changes in the number of GABA_A_ receptors suggests that modification of GABA_A_ receptors by phosphorylation can lead to the subsequent increase of the receptor conductance, thereby resulting in PM-LTP. Because the treatment with PKA inhibitors, H-89 and cAMPS-Rp, completely blocked the induction of PM-LTP, PKA is likely responsible for phosphorylation of GABA_A_ receptors to induced PM-LTP. Indeed, numerous studies reported that PKA directly phosphorylates β subunit of GABA_A_ receptors [36–39]. Furthermore, at interneuron-PC GABAergic synapses in the cerebellar cortex, activation of PKA facilitates both IPSCs and current responses to exogenous GABA [40]. To date, among the metabotropic purinergic receptors that have been investigated, the activation of P2Y_11_ subtype receptor and the associated G_s_ protein activates PKA [41, 42]. ATP is an endogenous agonist for P2Y_11_ receptors [43], and P2Y_11_ receptors have been demonstrated to localize in PCs of rat cerebellar cortex [12, 44]. Our data therefore suggest that ATP binding to P2Y_11_ receptors activates PKA, which, in turn, phosphorylates GABA_A_ receptors to increase the receptor conductance and thereby induces PM-LTP (Fig 5).

In the presence of the PKA inhibitor H-89, ATP transiently suppressed eIPSCs (see Fig 4A). The observation could be explained by the two possibilities as follows: (1) ATP may act on adenosine A_1_ receptors and suppress GABA release from BC terminals [45]; and (2) ATP can increase the frequency of sIPSCs, thereby causing fatigue of the GABA release machinery that would lead to suppression of eIPSCs [10]. The fact that ATP did not affect the eIPSC amplitude in the presence of cAMPS-Rp (see Fig 4B) might be consistent with the notion that cAMPS-Rp, an analog of cAMP, may activate hyperpolarization-activated cyclic nucleotide-gated (HCN) channels of BCs [46, 47] and thereby counterbalance the inhibitory action of adenosine.

It remains, however, to be determined whether or not PM-LTP is exclusively mediated by activation of PKA, because the activation of P2Y_11_ receptor may concurrently trigger intracellular Ca^2+^ release from endoplasmic reticulum via activation of the signaling cascade that includes associated G_q/11_ protein, phospholipase C (PLC) and inositol 1, 4, 5-trisphosphate [44]. Indeed, Saitow *et al*. [10] reported that P2Y_1_ receptor-mediated increase of intracellular Ca^2+^ can induce PM-LTP. In this study, inhibition of CaMKII did not suppress the potentiation of eIPSC during the period 10 to 15 min after ATP application, whereas the amplitude of eIPSCs thereafter reversibly recovered to the control level. The time course of eIPSC amplitudes after treatment with the CaMKII inhibitor (Fig 4C) was markedly different from those recorded in the experiment in which ATP alone was applied (Fig 2A): the ATP-induced increase in the amplitude of eIPSCs lasted for more than 20 min in the absence of KN-62. These results suggest that ATP-induced PM-LTP has at least two, PKA-dependent and CaMKII-dependent, components: the induction of PKA-dependent process appeared to be much faster than that of CaMKII-dependent process. The latter component may also be mediated by other subtypes of metabotropic purinoceptors on PCs, such as P2Y_1_, P2Y_2_, P2Y_4_, and P2Y_6_ receptors, because activation of those receptors triggers PLC to mobilize intracellular Ca^2+^ [43]. Further studies using subtype-specific purinergic agonists and antagonists are needed to determine particular subtypes of purinoceptors that may contribute to PM-LTP. Another possible component that might affect PM-LTP is the activation of protein kinase C (PKC), downstream of increased intracellular Ca^2+^, which can also directly phosphorylate β subunit of GABA_A_ receptors. However, the activation of PKC is reported to suppress GABA_A_ receptor currents in the cultured cortical neuron [48].

## Conclusions

The present study demonstrates that PS-NSFA is useful to evaluate modulation of GABAergic transmission between cerebellar interneurons and PCs. Our data reveal that there are at least two separate forms of long-term potentiation at the GABAergic synapse on PCs through the increases in either the number or the single-channel conductance intensity of postsynaptic GABA_A_ receptors, which are mediated by separate signaling pathways involving different second messengers. Such distinct forms of synaptic plasticity could be beneficial for fine tuning of the output from PCs that is responsible for the regulatory mechanism underlying motor function and learning.

## References

[1] Kano M, Rexhausen U, Dreessen J, Konnerth A. Synaptic excitation produces a long-lasting rebound potentiation of inhibitory synaptic signals in cerebellar Purkinje cells. Nature 1992; 356: 601–604. 1313949 10.1038/356601a0

[2] Llano I, Leresche N, Marty A. Calcium entry increases the sensitivity of cerebellar Purkinje cells to applied GABA and decreases inhibitory synaptic currents. Neuron 1991; 6: 565–574. 2015092 10.1016/0896-6273(91)90059-9

[3] Hashimoto T, Ishii T, Ohmori H. Release of Ca^2+^ is the crucial step for the potentiation of IPSCs in the cultured cerebellar Purkinje cells of the rat. J Physiol 1996; 497: 611–627. 9003548 10.1113/jphysiol.1996.sp021794 PMC1160959

[4] Barria A, Muller D, Derkach V, Griffith LC, Soderling TR. Regulatory phosphorylation of AMPA-type glutamate receptors by CaMKII during long-term potentiation. Science 1997; 276: 2042–2045. 9197267 10.1126/science.276.5321.2042

[5] Benke TA, Lüthi A, Isaac JT, Collingridge GL. Modulation of AMPA receptor unitary conductance by synaptic activity. Nature 1998; 393: 793–797. 9655394 10.1038/31709

[6] Hayashi Y, Shi SH, Esteban JA, Piccini A, Poncer JC, Malinow R. Driving AMPA receptors into synapses by LTP and CaMKII: requirement for GluR1 and PDZ domain interaction. Science 2000; 287: 2262–2267. 10731148 10.1126/science.287.5461.2262

[7] Boehm J, Malinow R. AMPA receptor phosphorylation during synaptic plasticity. Biochemical Society Transactions 2005; 33: 1354–1356. 16246117 10.1042/BST0331354

[8] Lüthi A, Wikström MA, Palmer MJ, Matthews P, Benke TA, Isaac J, et al Bi-directional modulation of AMPA receptor unitary conductance by synaptic activity. BMC Neuroscience 2004; 5: 44 15538948 10.1186/1471-2202-5-44 PMC535344

[9] Brockhaus J, Dressel D, Herold S, Deitmer JW. Purinergic modulation of synaptic input to Purkinje neurons in rat cerebellar brain slices. Eur J Neurosci 2004; 19: 2221–2230. 15090048 10.1111/j.0953-816X.2004.03325.x

[10] Saitow F, Murakoshi T, Suzuki H, Konishi S. Metabotropic P2Y purinoceptor-mediated presynaptic and postsynaptic enhancement of cerebellar GABAergic transmission. J Neurosci 2005; 25: 2108–2116. 15728851 10.1523/JNEUROSCI.4254-04.2005 PMC6726053

[11] Deitmer JW, Brockhaus J, Casel D. Modulation of synaptic activity in Purkinje neurons by ATP. Cerebellum 2006; 5: 49–54. 16527764 10.1080/14734220500497456

[12] Donato R, Rodrigues RJ, Takahashi M, Tsai MC, Soto D, Miyagi K, et al GABA release by basket cells onto Purkinje cells, in rat cerebellar slices, is directly controlled by presynaptic purinergic receptors, modulating Ca^2+^ influx. Cell Calcium 2008; 44: 521–532. 10.1016/j.ceca.2008.03.006 18468677

[13] Robinson HP, Sahara Y, Kawai N. Nonstationary fluctuation analysis and direct resolution of single channel currents at postsynaptic sites. Biophys J 1991; 59: 295–304. 1706951 10.1016/S0006-3495(91)82223-8 PMC1281146

[14] Brickley SG, Cull-Candy SG, Farrant M. Single-channel properties of synaptic and extrasynaptic GABA_A_ receptors suggest differential targeting of receptor subtypes. J Neurosci 1999; 19: 2960–2973. 10191314 10.1523/JNEUROSCI.19-08-02960.1999 PMC6782265

[15] Jo YH, Schlichter R. Synaptic corelease of ATP and GABA in cultured spinal neurons. Nat Neurosci 1999; 2: 241–245. 10195216 10.1038/6344

[16] Jo YH, Role LW. Coordinate release of ATP and GABA at in vitro synapses of lateral hypothalamic neurons. J Neurosci 2002; 22: 4794–4804. 12077176 10.1523/JNEUROSCI.22-12-04794.2002 PMC6757749

[17] Piet R, Jahr CE. Glutamatergic and purinergic receptor-mediated calcium transients in Bergmann glial cells. J Neurosci 2007; 27: 4027–4035. 17428980 10.1523/JNEUROSCI.0462-07.2007 PMC2671228

[18] Metea MR, Newman EA. Calcium signaling in specialized glial cells. Glia 2006; 54: 650–655. 17006893 10.1002/glia.20352 PMC2289783

[19] Dityatev A, Rusakov DA. Molecular signals of plasticity at the tetrapartite synapse. Curr Opin Neurobiol 2011; 21: 353–359. 10.1016/j.conb.2010.12.006 21277196 PMC3368316

[20] Agulhon C, Sun MY, Murphy T, Myers T, Lauderdale K, Fiacco TA. Calcium signaling and gliotransmission in normal vs. reactive astrocytes. Front Pharmacol 2012; 3: 139 10.3389/fphar.2012.00139 22811669 PMC3395812

[21] Traynelis SF, Silver RA, Cull-Candy SG. Estimated conductance of glutamate receptor channels activated during EPSCs at the cerebellar mossy fiber-granule cell synapse. Neuron 1993; 11: 279–289. 7688973 10.1016/0896-6273(93)90184-s

[22] De Koninck Y, Mody I. Noise analysis of miniature IPSCs in adult rat brain slices: properties and modulation of synaptic GABA_A_ receptor channels. J Neurophysiol 1994; 71: 1318–1335. 8035217 10.1152/jn.1994.71.4.1318

[23] Silver RA, Cull-Candy SG, Takahashi T. Non-NMDA glutamate receptor occupancy and open probability at a rat cerebellar synapse with single and multiple release sites. J Physiol 1996; 494: 231–250. 8814618 10.1113/jphysiol.1996.sp021487 PMC1160626

[24] Silver RA, Momiyama A, Cull-Candy SG. Locus of frequency-dependent depression identified with multiple-probability fluctuation analysis at rat climbing fibre-Purkinje cell synapses. J Physiol 1998; 510: 881–902. 9660900 10.1111/j.1469-7793.1998.881bj.x PMC2231069

[25] Benke TA, Lüthi A, Palmer MJ, Wikström MA, Anderson WW, Isaac JT, et al Mathematical modelling of non-stationary fluctuation analysis for studying channel properties of synaptic AMPA receptors. J Physiol 2001; 537: 407–420. 11731574 10.1111/j.1469-7793.2001.00407.x PMC2278972

[26] Momiyama A, Silver RA, Hausser M, Notomi T, Wu Y, Shigemoto R, et al The density of AMPA receptors activated by a transmitter quantum at the climbing fibre-Purkinje cell synapse in immature rats. J Physiol 2003; 549: 75–92. 12665613 10.1113/jphysiol.2002.033472 PMC2342931

[27] Casel D, Brockhaus J, Deitmer JW. Enhancement of spontaneous synaptic activity in rat Purkinje neurones by ATP during development. J Physiol 2005; 568:111–22. 16002445 10.1113/jphysiol.2005.091371 PMC1474765

[28] Kano M, Kano M, Fukunaga K, Konnerth A. Ca(2+)-induced rebound potentiation of gamma-aminobutyric acid-mediated currents requires activation of Ca2+/calmodulin-dependent kinase II. Proc Natl Acad Sci USA 1996; 93: 13351–13356. 8917594 10.1073/pnas.93.23.13351 PMC24096

[29] Kawaguchi SY, Hirano T. Signaling cascade regulating long-term potentiation of GABA(A) receptor responsiveness in cerebellar Purkinje neurons. J Neurosci 2002; 22: 3969–3976. 12019316 10.1523/JNEUROSCI.22-10-03969.2002 PMC6757657

[30] Machu TK, Firestone JA, Browning MD. Ca^2+^/calmodulin-dependent protein kinase II and protein kinase C phosphorylate a synthetic peptide corresponding to a sequence that is specific for the gamma 2L subunit of the GABA_A_ receptor. J Neurochem 1993; 61: 375–377. 8390566 10.1111/j.1471-4159.1993.tb03582.x

[31] McDonald BJ, Moss SJ. Differential phosphorylation of intracellular domains of gamma-aminobutyric acid type A receptor subunits by calcium/calmodulin type 2-dependent protein kinase and cGMP-dependent protein kinase. J Biol Chem 1994; 269: 18111–18117. 8027073

[32] Kawaguchi SY, Hirano T. Sustained structural change of GABA(A) receptor-associated protein underlies long-term potentiation at inhibitory synapses on a cerebellar Purkinje neuron. J Neurosci 2007; 27: 6788–6799. 17581966 10.1523/JNEUROSCI.1981-07.2007 PMC6672699

[33] Chen ZW, Olsen RW. GABAA receptor associated proteins: a key factor regulating GABA_A_ receptor function. J Neurochem 2007; 100: 279–294. 17083446 10.1111/j.1471-4159.2006.04206.x

[34] Thomas P, Mortensen M, Hosie AM, Smart TG. Dynamic mobility of functional GABA_A_ receptors at inhibitory synapses. Nat Neurosci 2005; 8: 889–897. 15951809 10.1038/nn1483

[35] Bannai H, Lévi S, Schweizer C, Inoue T, Launey T, Racine V, et al Activity-dependent tuning of inhibitory neurotransmission based on GABA_A_R diffusion dynamics. Neuron 2009; 62: 670–682. 10.1016/j.neuron.2009.04.023 19524526

[36] Ymer S, Schofield PR, Draguhn A, Werner P, Köhler M, Seeburg PH. GABA_A_ receptor beta subunit heterogeneity: functional expression of cloned cDNAs. EMBO J 1989; 8: 1665–1670. 2548852 10.1002/j.1460-2075.1989.tb03557.x PMC401007

[37] Browning MD, Bureau M, Dudek EM, Olsen RW. Protein kinase C and cAMP-dependent protein kinase phosphorylate the beta subunit of the purified gamma-aminobutyric acid A receptor. Proc Natl Acad Sci USA 1990; 87: 1315–1318. 2154739 10.1073/pnas.87.4.1315 PMC53465

[38] Kirkness EF, Bovenkerk CF, Ueda T, Turner AJ. Phosphorylation of gamma-aminobutyrate (GABA)/benzodiazepine receptors by cyclic AMP-dependent protein kinase. Biochem J 1989; 259: 613–616. 2541695 10.1042/bj2590613 PMC1138555

[39] Swope SL, Moss SJ, Blackstone CD, Huganir RL. Phosphorylation of ligand-gated ion channels: a possible mode of synaptic plasticity. FASEB J 1992; 6: 2514–2523. 1375568

[40] Kano M, Konnerth A. Potentiation of GABA-mediated currents by cAMP-dependent protein kinase. Neuroreport 1992; 3: 563–566. 1421107 10.1097/00001756-199207000-00004

[41] Communi D, Govaerts C, Parmentier M, Boeynaems JM. Cloning of a human purinergic P2Y receptor coupled to phospholipase C and adenylyl cyclase. J Biol Chem 1997; 272: 31969–31973. 9405388 10.1074/jbc.272.51.31969

[42] Torres B, Zambon AC, Insel PA. P2Y11 receptors activate adenylyl cyclase and contribute to nucleotide-promoted cAMP formation in MDCK-D(1) cells. A mechanism for nucleotide-mediated autocrine-paracrine regulation. J Biol Chem 2002; 277: 7761–7765. 11788591 10.1074/jbc.M110352200

[43] Fischer W, Krügel U. P2Y receptors: focus on structural, pharmacological and functional aspects in the brain. Curr Med Chem 2007; 14: 2429–2455. 17979698 10.2174/092986707782023695

[44] Volonté C, Amadio S, D'Ambrosi N, Colpi M, Burnstock G. P2 receptor web: complexity and fine-tuning. Pharmacol Ther 2006; 112: 264–280. 16780954 10.1016/j.pharmthera.2005.04.012

[45] Konishi S, Mitoma H. Cyclic AMP-dependent and independent mechanisms for presynaptic modulation of GABAergic transmission in the cerebellar cortex In: Okada Y. editor. The Role of Adenosine in the Nervous System. Amsterdam: Elsevier Science B.V.: 1997 pp. 89–95.

[46] Saitow F, Konishi S. Excitability increase induced by β-adrenergic receptor-mediated activation of hyperpolariztion-activated cation channels in rat cerebellar basket cells. J Neurophysiol 2000; 84: 2026–2034. 11024095 10.1152/jn.2000.84.4.2026

[47] Southan AP, Morris NP, Stephen GJ, Robertson B. Hyperpolarization-activated currents in presynaptic terminals of mouse cerebellar basket cells. J Physiol 2000; 526: 91–97. 10878102 10.1111/j.1469-7793.2000.t01-1-00091.x PMC2270001

[48] Brandon NJ, Delmas P, Kittler JT, McDonald BJ, Sieghart W, Brown DA, et al GABA_A_ receptor phosphorylation and functional modulation in cortical neurons by a protein kinase C-dependent pathway. J Biol Chem 2000; 275: 38856–38862. 10978327 10.1074/jbc.M004910200

